# Clostridium butyricum, a future star in sepsis treatment

**DOI:** 10.3389/fcimb.2024.1484371

**Published:** 2024-12-06

**Authors:** Jinglin Zhao, Li Jiang, Weizhi He, Dingrui Han, Xuan Yang, Liuli Wu, Haiyan Zhong

**Affiliations:** ^1^ Medical Laboratory, Kunming Children’s Hospital, Children’s Hospital Affiliated to Kunming Medical University, Kunming, Yunnan, China; ^2^ The Affiliated Hospital of Kunming University of Science and Technology, The First People’s Hospital of Yunnan Province, Kunming, China

**Keywords:** sepsis, *Clostridium butyricum*, probiotics, treatment, gut microbiota

## Abstract

Sepsis is a systemic inflammatory response syndrome of multiorgan failure caused by dysregulation of the host response to infection and is a major cause of death in critically ill patients. In recent years, with the continuous development of sequencing technology, the intestinal microecology of this disease has been increasingly studied. The gut microbiota plays a host-protective role mainly through the maintenance of normal immune function and the intestinal barrier. Recent evidence suggests that intestinal flora dysbiosis plays a crucial role in sepsis. *Clostridium butyricum (C. butyricum)*, which has been used as a probiotic in poultry feed since its discovery, has been found to play a potential protective role in intestinal infections, inflammatory bowel disease (IBD), colorectal cancer, and other diseases in recent studies. In this review, we continue to focus on the important role and mechanism of *C. butyricum* as a probiotic in human diseases, especially intestinal diseases. Additionally, we evaluate the research progress of *C. butyricum* in treatment of sepsis to identify more therapeutic targets for the clinical treatment of sepsis.

## 1 Introduction

Sepsis is a systemic inflammatory response syndrome caused by various inflammatory factors that can involve multiple organs and has high morbidity and mortality rates (Salomão et al., 2019). It is estimated that sepsis affects approximately 30 million people globally each year, with more than 6 million deaths and a patient mortality rate of up to 30% (Srzić et al., 2022; Oczkowski et al., 2022). The current treatment for sepsis mainly consists of treating the infection with fluids and organ support and controlling the source of infection for appropriate resuscitation (Liu et al., 2022; Zampieri et al., 2023; Vincent, 2022). Although current treatment methods can alleviate the occurrence of septic shock to a certain extent, there is no significant improvement in the morbidity and sepsis remains a major threat to human health.

**Figure 1:**
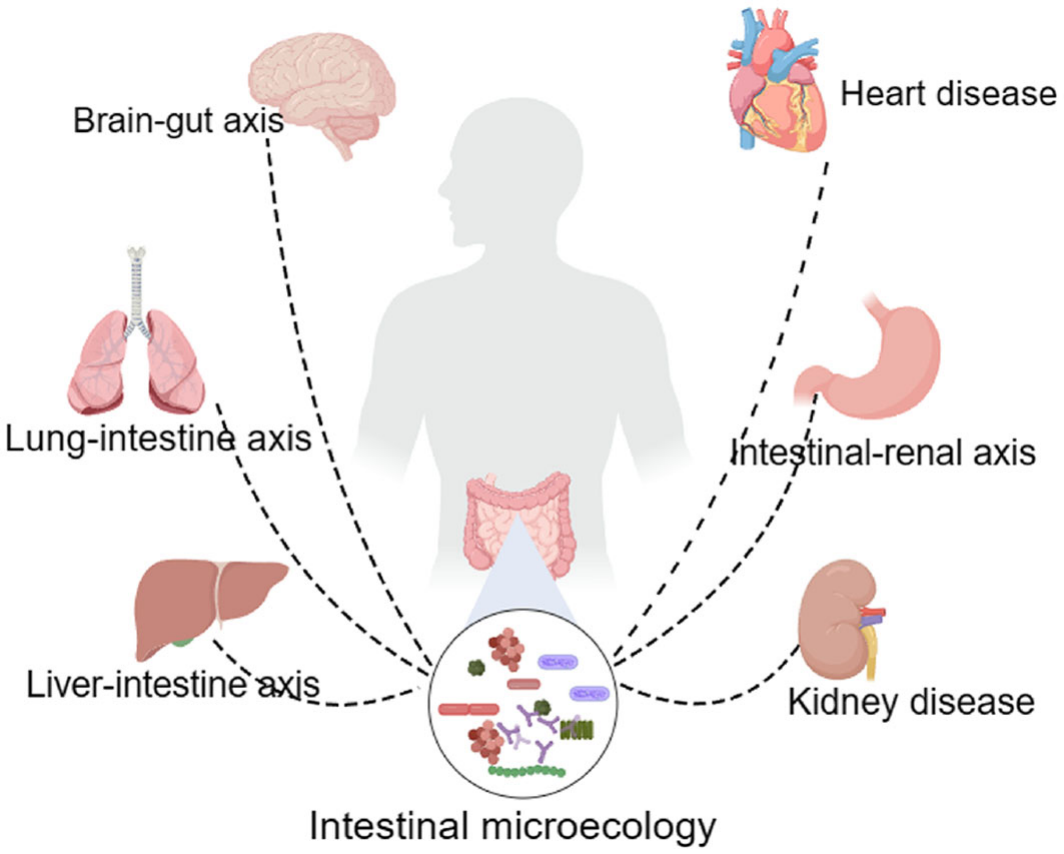
Intestinal microbiota affect multiple tissues and organs in the human body.

An increasing number of studies have shown that dysregulation is an important factor in sepsis and further accelerates the transformation of sepsis to septic shock (Xu et al., 2022; Yang et al., 2023). The intestinal microbiota of organisms can be broadly categorized into three groups, the first of which is the commensal flora, which mainly consists of *Bacteroides*, *Clostridium*, *Bifidobacterium*, and *Lactobacillus* (Yan and Wu, 2023). These bacteria account for more than 99% of the intestinal flora, form a good cooperative relationship with the host, assist in the digestion of a variety of foods, synthesize vitamins to enhance the body’s immunity, and maintain the ecological balance of the intestinal flora (Guo et al., 2020). The second category includes conditionally pathogenic flora, mainly *enterococci* and *enterobacteria* (Bowen et al., 2018). The number of this type of flora is not large, but it is unstable in the intestinal tract. When an organism is stimulated or the host’s immunity is low, this type of bacteria can transform into pathogenic bacteria, causing severe damage to the organism (Bowen et al., 2018; Kwiecinski and Horswill, 2020). The third category is pathogenic groups of bacteria, such as *Salmonella* and *pathogenic E. coli*. When these pathogenic bacteria enter the body and multiply, they cause a series of intestinal reactions in the body, leading to severe damage (Bäumler and Sperandio, 2016). Studies have shown (Haak and Wiersinga, 2017; Wang et al., 2019) that during sepsis, the intestinal microecosystem is significantly imbalanced, the beneficial flora of the body, such as *Bacillus, Clostridium, Bifidobacterium* and *Lactobacillus*, are significantly reduced, and the abundance of pathogenic bacteria is significantly increased (Xiao et al., 2023). This imbalance further exacerbates the damage to the intestinal barrier, causing pathogenic bacteria to enter the bloodstream and lymphatic system and spread throughout the body, leading to multiple organ failure, ultimately resulting in septic shock and death of the body (Chen et al., 2023; Deng et al., 2021) (**Figure 1**).

**Figure 2:**
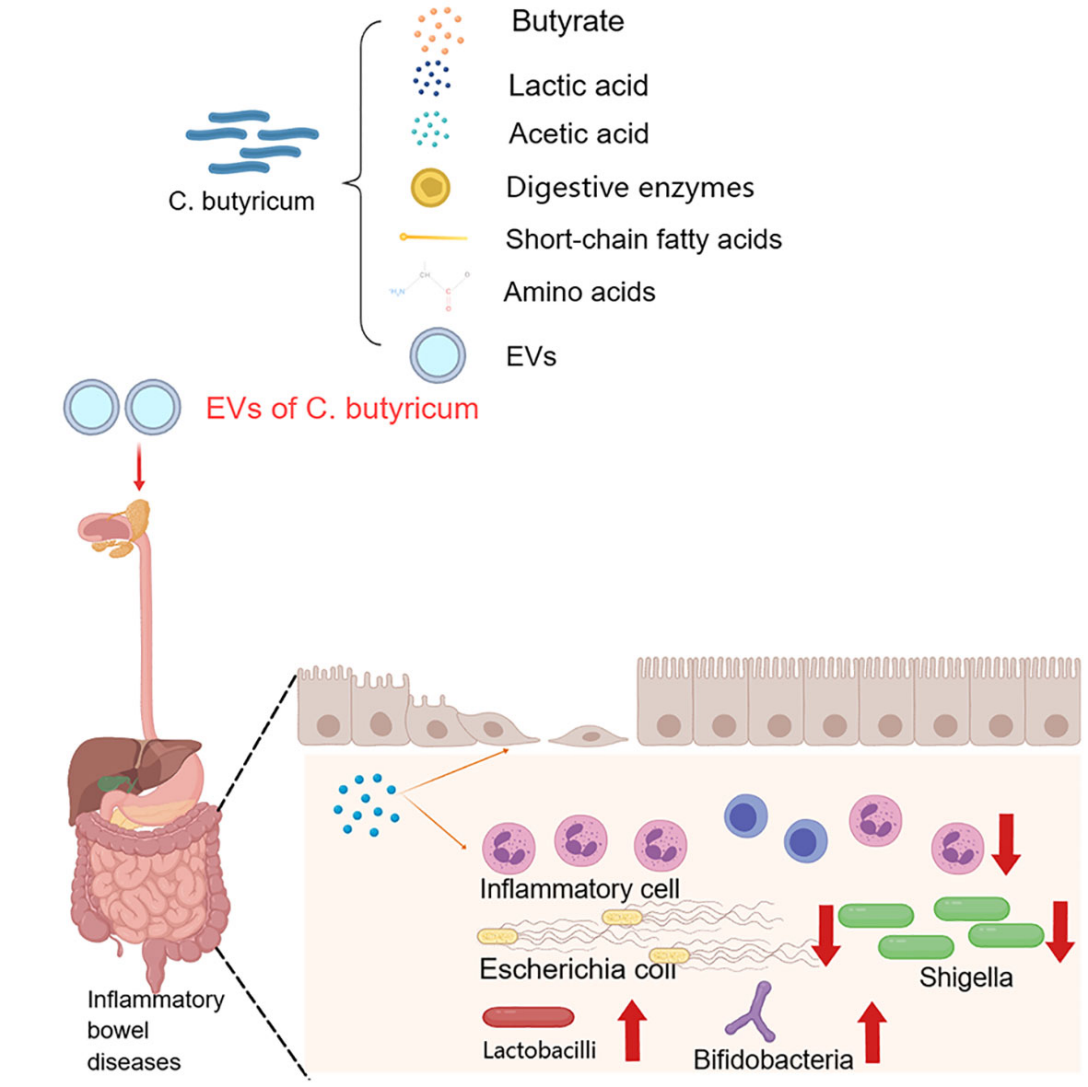
The effect of *Clostridium butyricum* on intestinal diseases and its possible mechanisms.

*C. butyricum*, also known as butyric acid bacteria, is an anaerobic bacillus with good tolerance to high temperature, high pressure and bile salts (Stoeva et al., 2021). Since its discovery by Dr. Konji Miyaji in Japan in 1933, research on *C. butyricum* has been gaining attention (Collins and East, 1998; Kanauchi et al., 2003). As a member of the Clostridium family, *C. butyricum* has a strong integumentary effect, inhibiting pathogenic bacteria in the intestinal tract and promoting the growth of beneficial bacteria in the intestinal tract, such as *bifidobacteria* and *lactobacilli* (Fenicia et al., 2002; Peck, 2009). On the one hand, the metabolites of *C. butyricum*, such as digestive enzymes, short-chain fatty acids (SCFAS) and amino acids, can protect the stability of the intestinal microecology and improve feed utilization (Cao et al., 2022). On the other hand, *C. butyricum* produces a large amount of butyric acid through metabolism to protect intestinal health and promote the repair and regeneration of intestinal epithelial cells (Pouillart, 1998). As a probiotic, *C. butyricum* has the ability to nourish the body, regulate the intestinal microecology, and enhance the immunity of the body; although the specific mechanism has not been fully elucidated, the available evidence suggests its potential as a promising clinical therapeutic candidate. Several studies have shown that *C. butyricum* has a certain preventive effect on inflammatory bowel disease (IBD), colon cancer (Budu et al., 2022; Liu et al., 2020), obesity (Obanda et al., 2020), diabetes (Lee et al., 2022) and other diseases; among them, the MIYAIRI-588 strain is widely used in Japan, China and other regions for the treatment of antibiotic-associated diarrhoea, and existing *C. butyricum* live-bacterial microecological preparations (Atenin, Misan, and Changlakang) have achieved some efficacy in the treatment of diarrhoea, IBD, and intestinal tumors (Zhang et al., 2023; Ariyoshi et al., 2020) (**Figure 2**). Therefore, in this narrative review, we focus on the specific mechanism of *C. butyricum* regulating intestinal microecology in various diseases and its current research in sepsis and analyze its application prospect in sepsis treatment in the future.

## 2 Review of the role of *C. butyricum* in intestinal diseases

### 2.1 *C. butyricum* regulates the mechanism of inflammatory bowel disease

Inflammatory bowel diseases (IBD), including ulcerative colitis and Crohn’s disease, are specific inflammatory lesions that occur in the rectum and colon (Adolph et al., 2022). Disruption of the intestinal epithelial barrier leads to increased permeability and infiltration of pathogens and further compromises the body’s immune system, leading to the development of IBD (Basso et al., 2014). An experimental animal study showed (Ma et al., 2022) that extracellular vesicle of C. *butyricum*-derived metabolites play an important role in alleviating colitis. Administering extracellular vesicles of *C. butyricum* to mice with IBD resulted in a dual effect; on the one hand, it reduced inflammatory cell infiltration and mucus layer damage in the colon, significantly enhancing the integrity of the intestinal mucus barrier; on the other hand, it modulated the composition of the intestinal microflora, significantly decreasing the abundance of intestinal pathogenic bacteria, such as *Shigella* and *Escherichia coli.* Transcriptome sequencing revealed a significant increase in tryptophan content in mice treated with extracellular vesicles, which play an important role in immunity, neuronal function, and intestinal homeostasis regulation. This finding suggested that extracellular vesicles of *C. butyricum* metabolites may be potentially important targets for the treatment of IBD. Moreover, in another study, *C. butyricum* was found to inhibit TLR2 signaling and IL-17 secretion and enhance the local mucosal immune system, thus exerting a protective effect against intestinal inflammation (Xie et al., 2020).

### 2.2 *C. butyricum* regulates intestinal tumor mechanisms

An increasing number of studies have also investigated the relationship between intestinal flora dysbiosis and the occurrence and development of intestinal tumors (Ge et al., 2021; Wong and Yu, 2019). In colorectal cancer patients, researchers found that *C. butyricum* significantly inhibited the development of intestinal tumors in APC-positive mice induced by a high-fat diet (HFD) (Zhou et al., 2017). In addition, mechanistic studies have shown that *C. butyricum* inhibits the Wnt/β-linker signaling pathway and modulates the composition of the gut microbiota, significantly reducing the abundance of pathogenic bacteria, such as *Escherichia coli* and *Shigella*, and increasing the content of probiotics, such as *Lactobacillus*, and their metabolites, such as free fatty acids (Chen et al., 2020). In another study (Zhou et al., 2022), FISH revealed that *C. butyricum* was mainly enriched in small intestinal crypts and colonic tumor tissues in a mouse model of colorectal cancer and may inhibit tumor progression by infiltrating into colonic tumor tissues and acting directly on colorectal cancer cells. Moreover, colorectal cancer cell lines were cocultured with *C. butyricum* to detect the expression of the MyD88 and Nuclear factor kappa-B (NF-κB) genes, and the expression of the two genes in the colorectal cancer cell lines was reduced by *C. butyricum* treatment, which suggested that *C. butyricum* may inhibit the progression of colorectal cancer by regulating the expression of the MyD88 and NF-κB genes.

In summary, *C. butyricum* plays an important role in various intestinal diseases, and its related molecular mechanism has also undergone preliminary exploration. Moreover, as *C. butyricum* is a probiotic, its corresponding products have been developed for the prevention and treatment of other diseases, such as improving the metabolic capacity of pancreas, adipose tissue, and liver to protect physical health (Li et al., 2023), restored the diminished efficacy of ICB and improved survival in lung cancer patients (Tomita et al., 2022). Resisting type 2 diabetes and reducing obesity (Stoeva et al., 2021). However, whether *C. butyricum* plays an important role in sepsis, as well as its potential mechanism, must be further investigated.

## 3 Specific role of *C. butyricum* in sepsis

### 3.1 Intestinal microecological changes in patients with sepsis

Sepsis is one of the most common serious illnesses in intensive care units. The pathogenesis of sepsis is extremely complex and mainly involves immune disorders, mitochondrial damage, coagulation disorders, inflammatory dysregulation, endoplasmic reticulum stress and autophagy (Gotts and Matthay, 2016; Jacobi, 2022). The intestinal flora also plays an important role in these processes. According to research reports, healthy intestinal flora can improve the immune function of sepsis patients, enhance host resistance, and prevent the occurrence of septic shock as well as systemic multiorgan failure syndrome (Gong et al., 2019; Sun et al., 2023). At the same time, healthy intestinal flora also produces beneficial metabolites (e.g.,SAFCs), regulate endoplasmic reticulum stress, improve multiorgan functional damage, change the intestinal microenvironment, enhance the body’s intestinal barrier function, prevent bacterial translocation, and so on. Compared with that of healthy people, the intestinal flora of patients with sepsis is significantly changed, mainly manifested by a decrease in the diversity of the intestinal flora, an increase in the abundance of a large number of pathogenic bacteria, such as *Escherichia coli* and *Shigella*, and a decrease in the abundance of beneficial bacteria, such as *Lactobacillus* (Cuna et al., 2021; Haussner et al., 2019). Moreover (Valdés-Duque et al., 2020) dysbiosis of the intestinal flora in sepsis patients further promotes the progression of sepsis to septic shock by decreasing the levels of metabolites, such as free fatty acids, and initiating the inflammatory immune response, which further leads to systemic multiorgan failure.

### 3.2 Intestinal regulation of *C. butyricum* in sepsis

**Figure 3:**
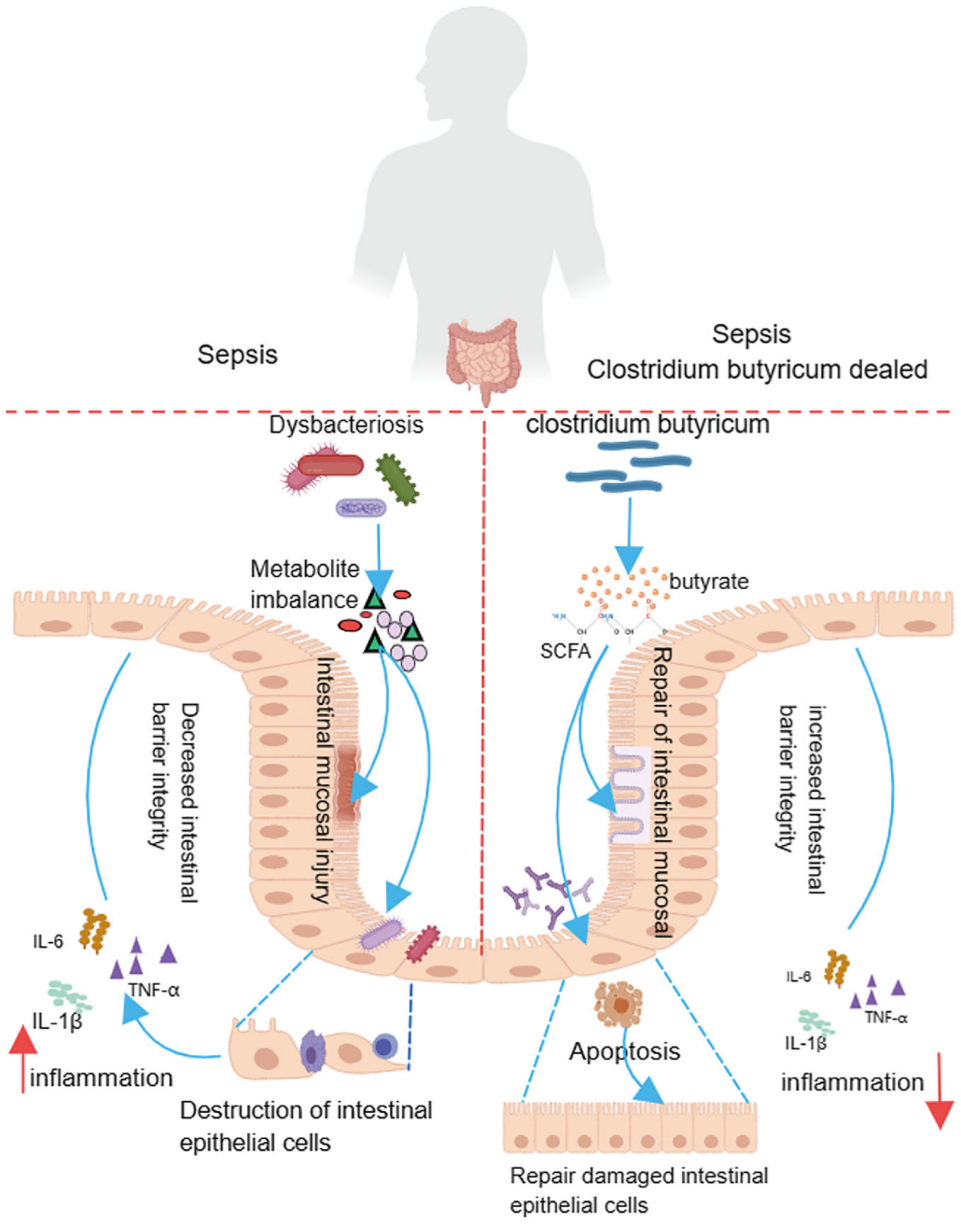
*Clostridium butyricum* plays an intestinal regulatory role in sepsis.

As a specialized anaerobic gram-positive bacillus, the metabolites of *C. butyricum* include SCFAS such as butyric acid, acetic acid, lactic acid, B vitamins, digestive enzymes and folic acid, which can regulate the balance of the intestinal flora, promote the proliferation of beneficial intestinal flora, inhibit the propagation of harmful bacteria, and have a strong rectification effect (Gao et al., 2023). Butyric acid, one of the most important metabolites of *C. butyricum*, can significantly stimulate intestinal epithelial papillary hyperplasia and villus growth and promote intestinal epithelial cell healing, and it has the greatest impact on the physiology and immunity of the human body through a variety of SCFAS (Bruce et al., 2014; Hsiao et al., 2021). Macfarlane CT et al. (Macfarlane and Macfarlane, 2011) showed that an organism is in a hypoxic state when sepsis occurs, and intestinal epithelium ischemia and anoxia lead to cellular damage and mucosal barrier disruption. Kripke et al. (1989). also found that after gavage of 40 mmol/ml butyric acid, the DNA content of the intestinal mucosal tissue was significantly greater in mice than in control mice. The DNA content of the mucosal tissues increased significantly, confirming that butyric acid can stimulate the proliferation of colonic mucosal epithelial cells and ameliorate the damage to intestinal tissues induced by sepsis. In another *in vivo* study in septic rats (Wang et al., 2015), it was found that compared to that in septic control rats, pathological damage to the intestinal mucosa in septic rats was reduced after butyrate treatment, and the expression of proinflammatory cytokine mRNA was significantly decreased. A further increase in the treatment dose revealed that a high dose of butyric acid significantly activated Caspase-1, elevated the expression of Caspase-1 and its shear bodies P20 and NLRP3, increased apoptosis in the intestinal lamina propria, and ameliorated sepsis-induced intestinal mucosal tissue injury (**Figure 3**). Moreover, a recent study from Japan also found a significant decrease in the abundance of Clostridiales genus on the first and seventh days of sepsis mouse models, and its corresponding gut microbiota metabolites such as SCFAS and butyric acid also decreased significantly (Muratsu et al., 2022).

### 3.3 Protective role of *C. butyricum* in sepsis

**Figure 4:**
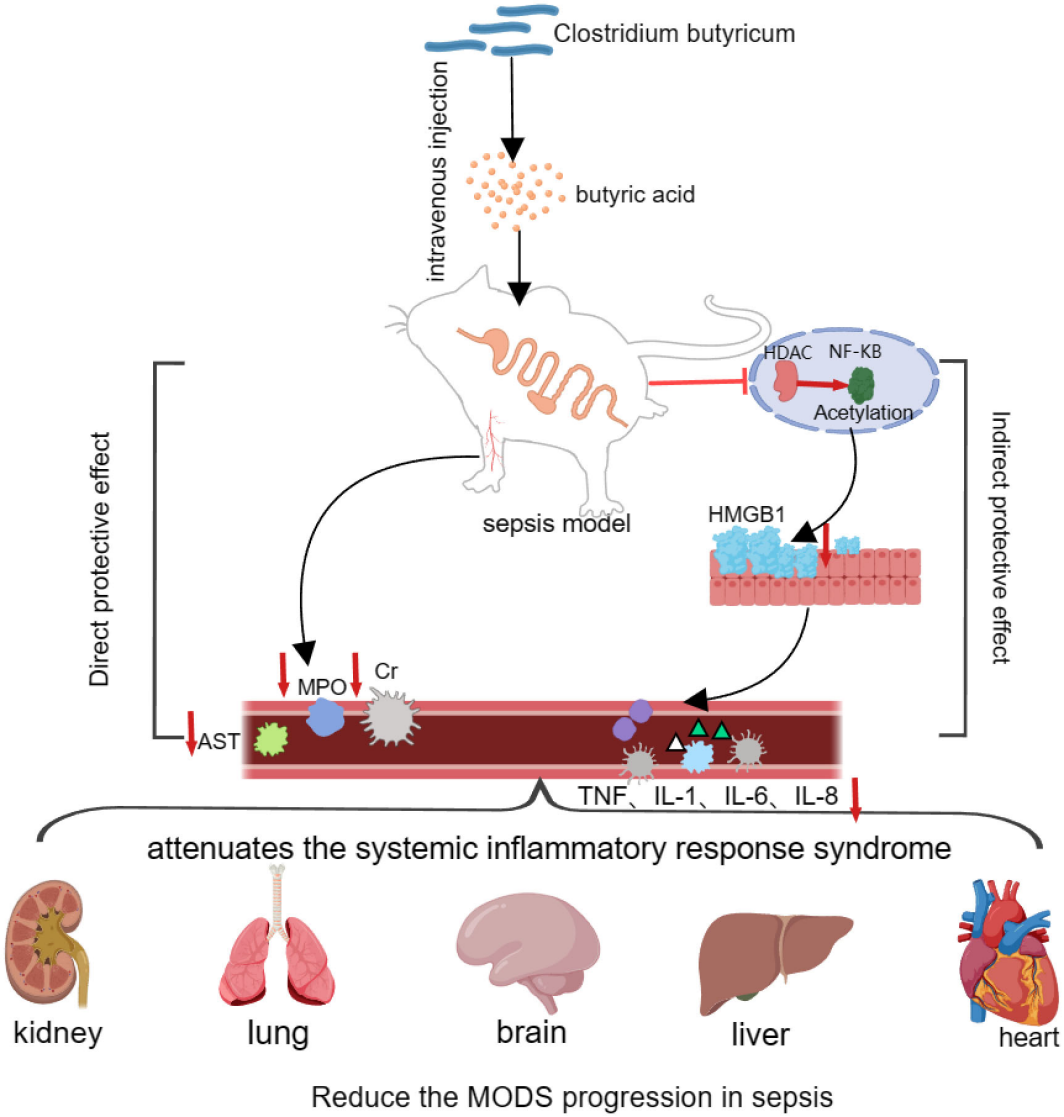
*Clostridium butyricum* plays an organ-protective role in sepsis.

In addition to its intestinal regulatory effects, butyric acid also has protective effects on septic organs. Animal studies have shown that intravenous injection of butyric acid in a rat Cecum Ligation AND Puncture (CLP) sepsis model can significantly reduce the mRNA level of HMGB1 in rat tissues, and the serum alanine aminotransferase (ALT), creatinine (Cre), and pulmonary peroxidase (MPO) levels were significantly lower in the CLP group than in the control group, which confirms that butyric acid can significantly alleviate systemic multiorgan failure caused by sepsis (Zhang et al., 2007). HMGB1, a member of the high mobility family of proteins, can be secreted to the extracellular level to participate in the activation of the inflammatory response and induce endothelial cells to express adhesion molecules for chemotaxis of inflammatory cells, such as tissue extravasation, thus aggravating the systemic inflammatory response syndrome of sepsis (Naglova and Bucova, 2012). Butyric acid, an inhibitor of the enzyme deacetylase (HDAC), can inhibit deacetylation to cause acetylation of the transcription factor NF-κB, thereby attenuating NF-κB-mediated systemic inflammatory response syndrome. NF-κB-mediated transcription of the HGMB1 gene ultimately reduces the mRNA level of HMGB1 and attenuates the systemic inflammatory response syndrome induced by sepsis (Quivy and Van Lint, 2004; Shirasugi et al., 2018). In a mouse model of sepsis brain injury, researchers found that compared with those in the sepsis group, the water maze-generated mice in the sodium butyrate pretreatment group exhibited a shorter escape latency and a greater number of shuttle platforms, which significantly improved neuronal degeneration in the hippocampus of the mice in the sepsis group and attenuated sepsis-induced brain injury in the mice (Bi et al., 2019; Lisman et al., 2017). Moreover, several studies have shown that in sepsis models, intraperitoneal injection of 200 mg/kg sodium butyrate can improve acute lung injury (Zhang et al., 2010), sepsis-induced myocardial inhibition (de Lazari et al., 2020), intestinal barrier function (Rivière et al., 2016), and other functions caused by sepsis. These findings further suggest that *C. butyricum* plays a modulating role in the treatment of sepsis (**Figure 4**).

### 3.4 Immunomodulatory role of *C. butyricum* in sepsis

In addition to its extremely strong rectifying effect, *C. butyricum* also plays an important role in the immunomodulatory function of sepsis. According to a previous study (Zhang et al., 2016; Xie et al., 2020), *C. butyricum* can activate dendritic cells and macrophages in the intestinal tract through the Toll-like receptor 2 (TLR2) pathway and promote the secretion of the transforming growth factor β (TGF-β), which further mediates the differentiation of initial T cells to regulatory T cells (Treg), and the interleukin 10 (IL-10) produced by Tregs inhibits the proinflammatory response mediated by Th1 and Th 17, thus exerting anti-inflammatory effects. Similarly, in another animal study, sepsis-related lung injury was established in mice by CLP and treated with butyrate gavage after surgery. The results of the study found that butyrate effectively improved the survival rate and alleviated lung damage in CLP mice, and the main mechanism may be that butyrate increased the number of CD4 + Foxp3 + Tregs and enhanced the barrier function of the gut and lungs (Wei et al., 2024).

This review also has several limitations. According to the literature, the protective effect of *C. butyricum* on sepsis is still in the basic research stage in animal models. Whether *C. butyricum* plays a protective role in humans remains to be fully explored in clinical experiments. We look forward to further research on the preventive and protective effects of *C. butyricum* on sepsis in the future, providing new insights for the treatment of sepsis patients.

## 4 Conclusion

Intestinal microecology is a new direction for treatment of sepsis. In view of the important role of *C. butyricum* in the maintenance of intestinal homeostasis and immune regulation, this review provides theoretical support for the future application of *C. butyricum* in treatment of sepsis, and we believe that *C. butyricum* could become a new star in treatment of sepsis in the future.
